# Research Data in Core Journals in Biology, Chemistry, Mathematics, and Physics

**DOI:** 10.1371/journal.pone.0143460

**Published:** 2015-12-04

**Authors:** Ryan P. Womack

**Affiliations:** Rutgers University Libraries, Rutgers-The State University of New Jersey, New Brunswick, New Jersey, United States of America; Universidad de Las Palmas de Gran Canaria, SPAIN

## Abstract

This study takes a stratified random sample of articles published in 2014 from the top 10 journals in the disciplines of biology, chemistry, mathematics, and physics, as ranked by impact factor. Sampled articles were examined for their reporting of original data or reuse of prior data, and were coded for whether the data was publicly shared or otherwise made available to readers. Other characteristics such as the sharing of software code used for analysis and use of data citation and DOIs for data were examined. The study finds that data sharing practices are still relatively rare in these disciplines’ top journals, but that the disciplines have markedly different practices. Biology top journals share original data at the highest rate, and physics top journals share at the lowest rate. Overall, the study finds that within the top journals, only 13% of articles with original data published in 2014 make the data available to others.

## Introduction

Recent years have seen an increased emphasis on the research data used to develop findings in published scholarly articles. Several related concepts have emerged, including data citation, data sharing, reproducibility, data publication, and open data. The current study will provide empirical evidence for the presence or absence of these practices in leading scientific journals in four fundamental disciplines: biology, chemistry, mathematics, and physics. First, these concepts are reviewed.

### Data citation

Data citation, meaning a direct link or reference to a data source, as distinct from citing an article that uses data, is increasingly recommended as a way of ensuring the discoverability and reusability of research data supporting published articles [1–3]. Enhanced data citation with machine-actionable links can support a more complex ecosystem of scholarship that makes a wider range of types of publications and discussions available [4]. DataCite (https://www.datacite.org) and other initiatives are encouraging the widespread adoption of digital object identifiers (DOIs) and standard citation practices for data.

### Data sharing

The trend towards greater emphasis on data sharing is driven by many influences [5]. Funders of research, including the major US agencies such as the National Science Foundation (NSF) and National Institutes of Health (NIH), are under increasing pressure to demonstrate the impact and relevance of their efforts, and are requiring data sharing in order to ensure transparency and reusability in research. Along with prior requirements for grant applicants to submit data management plans, the recent agency responses to the White House’s Office of Science and Technology Policy mandate strong efforts to make research data that results from federal funding publicly available. A compilation of these responses is available at http://guides.library.oregonstate.edu/federaloa. Publishers and researchers are also seeking to maximize the visibility, influence, and impact of their research products by extending availability from the written article to the associated data behind the publication. When data is expensive to gather or unique, its preservation and availability to other researchers is essential to encourage scientific progress. Since virtually all astronomical, climatological, and biological observational data represents a capture of a unique, historical window of time, the amount of data that falls into this category is quite large.

In many disciplines, major repositories have been created to share data that is of common interest to the research community, such as the Protein Data Bank (http://www.rcsb.org/) or the Planetary Data System (https://pds.jpl.nasa.gov/). As one example, Williams describes disciplinary efforts to create data sharing repositories in chemistry [6]. As big science paradigms that use large heterogeneous data sources across large research teams are increasingly important in scientific research, direct and automated access to underlying research data sets is becoming even more significant, and many of the data repositories have been created to support these needs [7]. The re3data service (http://www.re3data.org) provides a searchable directory of major data repositories.

Also, more and more journals themselves are taking steps to make data available. One notable example is the Public Library of Science (PLoS) policy introduced in 2014, which requires authors to state where the data associated with the research can be accessed [8]. Regulative pressure from journals to make data available, via a data sharing policy or other methods, has been found to strongly influence author behaviors [9].

Scientists are gradually adopting their own data-sharing practices, in spite of challenges [10, 11]. At the same time, some may be reluctant to share data, although this could be related to the weakness of the authors’ findings [12]. Attitudes towards sharing may differ by faculty rank [13]. Fecher documents the ongoing structural barriers in attitudes to data sharing and argues for the need to greater incentivize data sharing via recognition and reimbursement [14]. Making data available only via direct contact with the author, the minimal requirement for most grant-funded research, has been found to be ineffective, while mandated data sharing alongside the published article greatly improves access to data [15]. The “contact the author” method is also associated with rapidly decreasing data availability over time [16].

Librarians also need to keep up with the developments in data sharing in order to support researchers who are adapting to the changing nature of data-driven research [17]. The entire scholarly ecosystem is affected by data sharing.

### Reproducibility

In addition to making raw data available, the software code developed to conduct analysis is often essential to being able to reproduce and extend research findings, and the inclusion of software as part of the complete research data output is increasingly encouraged [18]. Reproducibility involves transparency about research methods and tools used, and is an important assurance of the reliability of the findings in any single publication.

### Data publication

Data publication refers to a more formal process of making data permanently available. The “data journal” has emerged as one vehicle for recognized data publishing, where data is released with an associated descriptive article. Nature’s *Scientific Data* (http:www.nature.com/sdata/) is a leading example, although repositories such as Dryad (http://datadryad.org) also serve a data publication role by working in partnership with journals to release data alongside articles. Institutional repositories at universities may also play a role in making data available in a reliable and discoverable manner. Data curation initiatives such as the Data Preservation Alliance for the Social Sciences (DATA-PASS) seek to preserve research data which may or may not be linked to article publication, placing emphasis on best practices for the long-term stewardship of the data [19].

While there is a lack of consensus on what constitutes data publication [20], work is ongoing to develop more extensive standards and criteria for data publication associated with journals, to ensure openness and reduce barriers to use [21], and to ensure the long-term stewardship of significant data [22].

### Open Data

Open data in the broad sense refers to data that is freely accessible, reusable, and sharable. This involves both public accessibility and usage rights that enable others to work with and build upon the data. The rights issues surrounding open data are an important part of making the data fully open [23]. This study will examine whether data is freely accessible, but does not delve into rights issues. None of the journal websites examined here explicitly describe the rights for available data on their associated download pages. Data sharing and data publication are steps toward fully open data, but may be incomplete without ensuring that the data is well-documented, formatted, and not subject to rights restrictions.

### Prior work

While an exhaustive literature review is not presented here, it is important to note a few prior studies of the availability of research data. Recognition of the issues involved in data sharing dates back decades, a notable example being the 1985 Report of the Committee on National Statistics, *Sharing Research Data* [24], along with discussion of the necessary transition to sharing via the Internet [25]. However, these studies did not attempt to directly measure the availability of data via examination of research output, an approach taken by more recent studies.

Nicholson and Bennett examined the availability of data in dissertations in selected disciplines [26]. They found that although two-thirds of the dissertations sampled had some data component that was made available, none of the dissertations in biology, sociology, mechanical engineering, or education made available the full raw data used to generate the dissertation. This is suggestive of patterns of data sharing that will be further examined in the current study.

Most closely related to the current study, in 2011 Alsheikh-Ali et. al. [27] examined papers published in 50 of the highest impact factor journals across all disciplines. Their study was a systematic sample, looking at the first 10 papers published in each journal, for a total sample size of 500. They found that although 88% of journals had instructions to authors about making data available, many articles were not subject to a data policy. Of those papers subject to a policy, most did not fully comply with the policy. Only 9% of the papers made available the complete research data associated with the article. This figure will be compared to the results of the current study. Other studies have examined data sharing in fields such as sociology [28] and genomics [29].

While the trend towards and the benefits of data sharing are clear, particularly for the advancement of statistical science, the process is by no means simple and getting to the goal of greater sharing requires settling many unresolved issues [30].

## Methods

### Objectives

The goal of the current study is to examine data use, data citation, and data sharing practices in leading journals in key scientific disciplines. Factors affecting reproducibility and openness, such as the free public availability of data and software code are also examined. The fundamental disciplines of biology, chemistry, mathematics, and physics were chosen because of their wide-ranging significance in science. For the purpose of the paper, data is defined as primarily numeric or raw measurement information that can be accessed in machine-actionable form. Since the intended purpose of data sharing is for other researchers to be able to make use of the information in their own work, data intended for illustration and observation only, such as videos, or tables reproduced only in PDF, are not coded as “data” in this study.

By using a fully randomized sampling method, statistically valid estimates of proportions of the variables of interest (such as the proportion of articles using and sharing data) can be generated, along with confidence intervals on the estimates. Treating the four disciplines as separate strata allows the use of stratified sampling techniques to combine the individual disciplinary estimates into a more precise estimate for the group of top journals as a whole. The primary emphasis of this study is on understanding the disciplinary patterns among the most influential journals, and does not, for the most part, seek to characterize the practices of individual journals. To study individual journal patterns in more detail would require larger sample sizes and a different approach to sampling. Here, sample sizes are limited due to time constraints on the analysis, and the methodology is chosen to derive useful information from the limited sample size. This study differs from other work in its focus on specific scientific disciplines and its use of statistical sampling techniques to generate more reliable parameter estimates.

### Definition of Target Population

The target population are “articles in leading scientific journals”, which are specified as follows. In each of four foundational science disciplines (biology, chemistry, mathematics, and physics), the top 10 journals are ranked according to the 5-year impact factor using the InCites Journal Citation Reports from Thomson Reuters [31].

The impact factor is a long-standing and well-recognized measure of the significance of a journal in scholarship, but there are certainly other ways to rank and assess the top journals in a discipline [32]. The impact factor measures the number of times a typical article in the journal is likely to be cited over time, by dividing total citations by the number of articles published in the journal. Although a high-volume publication with a low impact factor may have more total citations, each individual article in it is less likely to be recognized. So articles from the high-impact factor journals can be viewed as the most influential for the discipline. While recognizing that other selection methods are possible, the current study focuses on the impact factor as the criterion for selecting the top 10 journals in each field. In particular, the five-year impact factor, which smoothes annual fluctuations over a longer term, is used to provide a more stable cohort of top journals. While discipline experts may have their own views about the most significant publications or prefer other ranking metrics, this selection method has the advantage of being reproducible, not subjective, and applicable across disciplines. The use of a top 10 rather than a selection based on the number of journals in the discipline (such as the top-ranked two or three percent of journals) is arbitrary, but avoids the subjectivity inherent in defining which journals belong to the discipline and which do not. Some of the issues with the journals that result will be discussed later in the paper.

InCites Journal Citation Reports provides two category schema, “Web of Science” and the “Essential Science Indicators”. Because the Web of Science categories are too fine-grained, we use the “Essential Science Indicators”, which allow us to look at broad categories such as physics, mathematics, and chemistry. The category used for biology is actually “biology and biochemistry”. We use the 5-year impact factor measure to smooth out short-term variations in the impact factor.

Using the InCites category schema “Essential Science Indicators”, the top 10 journals ranked by the 5-year Impact Factor from Journal Citation Reports using 2013 data (the latest available at the time of the research) are shown in Tables 1, 2, 3 and 4.

**Table 1:** Biology top 10 journals by Impact Factor, 2013.

*rank*	*Journal*	*Total Cites*	*Impact Factor*	*5-year Impact Factor*
1	Nature Biotechnology	42,156	39.080	35.620
2	Physiological Reviews	23,974	29.041	35.456
3	Annual Review of Biochemistry	20,070	26.534	32.970
4	Nature Methods	24,560	25.953	27.195
5	Endocrine Reviews	13,623	19.358	24.124
6	Annual Review of Physiology	8,246	14.696	18.785
7	Annual Review of Biophysics	1,975	12.250	16.430
8	Nature Chemical Biology	12,495	13.217	15.059
9	Nature Protocols	20,399	7.782	13.142
10	PLOS Biology	24,324	11.771	12.807

**Table 2:** Chemistry top 10 journals by Impact Factor, 2013.

*rank*	*Journal*	*Total Cites*	*Impact Factor*	*5-year Impact Factor*
1	Chemical Reviews	124,463	45.661	48.832
2	Progress in Polymer Science	17,446	26.854	34.000
3	Chemical Society Reviews	63,071	30.425	33.159
4	Accounts of Chemical Research	47,005	24.348	26.002
5	Nature Chemistry	12,440	23.297	24.537
6	Acta Crystallographica Sect. A	12,476	2.069	17.237
7	Annual Review of Physical Chemistry	7,570	15.678	15.500
8	J. of Photochemistry & Photobiology C	2,239	11.625	14.424
9	ACS Nano	58,446	12.033	13.774
10	Aldrichimica Acta	1,066	16.333	13.667

**Table 3:** Mathematics top 10 journals by Impact Factor, 2013.

*rank*	*Journal*	*Total Cites*	*Impact Factor*	*5-year Impact Factor*
1	SIAM Review	5,484	4.791	9.833
2	J. Royal Statistical Society Series B	14,568	5.721	6.016
3	Annals of Statistics	13,953	2.442	4.209
4	Acta Mathematica	3,096	3.033	4.185
5	Appl. and Comp. Harmonic Analysis	2,086	3.000	3.904
6	J. of the American Mathematical Society	2,398	3.061	3.713
7	Annals of Mathematics	8,926	2.822	3.478
8	Foundations of Computational Mathematics	706	2.152	3.423
9	Statistical Science	3,503	1.690	3.411
10	Communications on Pure and Applied Math.	6,904	3.080	3.373

**Table 4:** Physics top 10 journals by Impact Factor, 2013.

*rank*	*Journal*	*Total Cites*	*Impact Factor*	*5-year Impact Factor*
1	Reviews of Modern Physics	37,647	42.860	52.577
2	Nature Photonics	18,623	29.958	32.342
3	Advances in Physics	5,026	18.062	27.921
4	Surface Science Reports	4,410	24.562	25.642
5	Physics Reports	21,386	22.910	25.010
6	Nature Physics	20,321	20.603	20.059
7	Nano Today	3,855	18.432	19.202
8	Living Reviews in Relativity	1,600	16.526	18.310
9	Advances in Optics and Photonics	660	9.688	18.194
10	Reports on Progress in Physics	11,421	15.633	16.627

### Sampling Methods

The sampling frame consists of all articles published in these 40 journals in 2014, the most recent complete year at the time of research. Selection and review of articles was conducted in March and April of 2015. Since we also want to assess the different patterns present in each discipline, we stratify by discipline and sample 50 articles for each discipline. The element and sampling unit is the journal article. BIOSIS was used to identify articles from the Biology journals. Web of Science, which covers all of the remaining top journals, was used to generate the remaining three groups of disciplinary listings.

The sample design is a stratified random sample. We stratify by discipline and not by journal, since each journal within the discipline will have different numbers of articles published and different data usage patterns. The intention is to gain an understanding of the overall pattern among influential journals in the discipline rather than to evaluate specific journals. This goal is reflected in our sampling method. While other more sophisticated and complex sampling schemes could be considered, this study presents no unusual issues in the nature of the data, or the difficulty and cost of conducting the survey, that would warrant a more complex design. In order to evaluate patterns at the journal level or to stratify at the journal level, a larger sample size would be required and a more complex formula to compute variances would be needed. Since this study’s sample size was limited by the time and resources available for the study, analysis beyond the disciplinary level is not feasible.

Using the population size of articles published for each discipline, a random listing of integers up to the maximum population size was generated for each group (using R software). The first 50 numbers in each group’s list were matched to the sequential list of articles generated by the search in the index to identify the articles selected in the sample. This method generates a probability of selection for each journal that is proportional to the number of articles published by the journal in that year. Therefore, journals that publish more articles are more likely to be selected in the sample. Articles were sampled without replacement.

Each article identified by the sampling process was downloaded in PDF form and was also examined on the publisher’s web portal for associated materials.

### Measurements

Articles were coded for the following primary characteristics:

- *Whether the article contained data or not*. If the article contained or used more than a trivial amount of data, it was considered a data article. If there was a reasonable expectation of some reader having a use for the underlying data, it was considered a data paper for the purpose of this article. For example, if a standard mathematical function was plotted using a limited amount of simulated or generated data, this was not considered a data paper, since an interested reader would not need the raw data to perform a similar task. But if a paper contained a mathematical algorithm whose validity was tested via a moderate amount of simulated data, this was considered a data paper, since an interested reader might want to test whether the author’s conclusions were peculiar to the particular data used. This initial coding is intended to reflect the use of data in the research for the article, regardless of how data is presented in the article itself. For example, if experimental data was presented in a graph, with no associated numeric tables or files, this was considered a data paper for the purposes of the study. In most cases the distinction between data papers and non-data papers was obvious.
- If the article contained data,
  - *Whether the data was original to the article* (i.e., generated by the research described in the article), or reused from other sources. If both original and reused data were present, the article was coded as having original data.
  - *Whether the data was available to the reader*, and if so, the method of access (journal, external site, other) and whether it was freely available or available only to subscribers. This study considers direct access to the data via links to be available data, and considers “contact the author” instructions as data that is not available.
- *Whether the article was a review article*. Review articles had distinctive characteristics that will be described later.
- *Whether a DOI or other citation method was provided linking directly to the data*, as distinct from the DOI or citation provided to the article itself.

In addition, notes were made on additional data-type products, such as videos or PDF documents containing tables. Each article was individually scanned for clearly labeled links to data in the relevant sections of the paper and in the references. All data was coded by the author. Since each paper was not scrutizinized word-for-word, and the author claims no special disciplinary expertise, errors in coding are possible, but there is no reason to believe that they would be pervasive or systematic. Availability of the coding worksheets and article references is described in the Supporting Information.

### Articles by Discipline

The selection procedure for articles is described in more detail in this section. The focus on the individual disciplines of biology, chemistry, mathematics, and physics, results in the exclusion of highly influential cross-disciplinary journals such as *Cell*, *Nature*, and *Science* from the sample. Also, a number of the high-impact journals in each discipline are review journals, which naturally have different characteristics since they are surveying existing research rather than reporting original findings.

One of the biology journals in the top 10 by impact factor, *Nature Protocols*, was not indexed by BIOSIS, so the author identified these 219 articles published in 2014 as a separate list appended to the list generated by BIOSIS for the purpose of the random sample. In other disciplines, the Web of Science index was able to generate a complete listing of all articles published in the journals in 2014. In retrospect, it would have been possible to use Web of Science to generate all of the biology sample, but the sample had already been collected via the supplemented BIOSIS list described above.

Categories of publication such as “addendum”, “corrigendum”, “editorial”, “news”, “correction”, “retraction”, and so on were excluded from consideration. Only items tagged by the index as “articles”, “reviews”, or, in the case of *Nature Protocols*, “protocols”, were considered part of the final sampling frame. The resulting numbers of articles are listed in Tables 5, 6, 7 and 8.

**Table 5:** Biology article counts, 2014.

*rank*	*Journal*	*items published*	*articles or reviews only*
1	Nature Biotechnology	129	103
2	Physiological Reviews	35	31
3	Annual Review of Biochemistry	31	31
4	Nature Methods	168	143
5	Endocrine Reviews	16	16
6	Annual Review of Physiology	28	28
7	Annual Review of Biophysics	20	19
8	Nature Chemical Biology	158	141
9	Nature Protocols	219	203
10	PLOS Biology	174	168
	Total for Biology	978	883

**Table 6:** Chemistry article counts, 2014.

*rank*	*Journal*	*items published*	*articles or reviews only*
1	Chemical Reviews	294	281
2	Progress in Polymer Science	67	64
3	Chemical Society Reviews	399	380
4	Accounts of Chemical Research	359	353
5	Nature Chemistry	256	133
6	Acta Crystallographica Sect. A	15	14
7	Annual Review of Physical Chemistry	28	27
8	J. of Photochemistry & Photobiology C	22	20
9	ACS Nano	1382	1328
10	Aldrichimica Acta	6	6
	Total for Chemistry	2828	2606

**Table 7:** Mathematics article counts, 2014.

*rank*	*Journal*	*items published*	*articles or reviews only*
1	SIAM Review	26	19
2	J. Royal Statistical Society Series B	39	38
3	Annals of Statistics	92	83
4	Acta Mathematica	15	15
5	Appl. and Comp. Harmonic Analysis	59	59
6	J. of the American Mathematical Society	26	26
7	Annals of Mathematics	46	46
8	Foundations of Computational Mathematics	39	38
9	Statistical Science	62	44
10	Communications on Pure and Applied Math.	49	46
	Total for Mathematics	453	414

**Table 8:** Physics article counts, 2014.

*rank*	*Journal*	*items published*	*articles or reviews only*
1	Reviews of Modern Physics	39	35
2	Nature Photonics	224	130
3	Advances in Physics	4	4
4	Surface Science Reports	11	11
5	Physics Reports	48	48
6	Nature Physics	273	131
7	Nano Today	56	35
8	Living Reviews in Relativity	7	7
9	Advances in Optics and Photonics	11	9
10	Reports on Progress in Physics	58	57
	Total for Physics	731	467

The population sizes of substantive articles and reviews published in 2014 for each discipline are Biology, 883; Chemistry, 2,606; Mathematics, 414; Physics, 467. Our total population of articles and reviews is therefore 4,370. We can see that within each discipline there are some journals that only publish a few articles a year, notably among the review journals, while some journals publish many more. *ACS Nano* dominates the chemistry sample with 50.9% of the articles. In fact, *ACS Nano* accounts for 30.4% of the entire population of articles in the sampling frame. When combined, *Nature Photonoics* and *Nature Physics* account for 55.9% of the physics sample. The four *Nature* titles, along with *PLoS Biology*, lead the biology sample in numbers of articles. Mathematics is more balanced, although *Annals of Statistics* leads in number of articles. It is important to keep these patterns in mind when interpreting the results, which only provide an overall picture of the top journals. The results do not portray the data availability at the journal level, only an estimate of the typical article published in the set of top journals.

## Results

We are primarily interested in the proportions of articles in several categories, so within each disciplinary category, we estimate the overall proportion by the sample proportion in Eq (1):

$$\begin{array}{c} \hat{p}=\frac{\sum_{i=1}^{n}y_{i}}{n} \end{array}$$ (1)

where *y*_*i*_ = 1 if the characteristic of interest is present, *N* is the population total (number of articles published in the top 10 disciplinary journals in 2014), and *n* is the sample size (50 for each of the four disciplines). Sampling formulae and methods used in this article follow Lohr’s *Sampling: Design and Analysis* [33]. The variance is then estimated by using the estimate of the proportion, according to Eq (2):

$$\begin{array}{c} \hat{V}\left(\hat{p}\right)=(1-\frac{n}{N})\frac{\hat{p}\left(1-\hat{p}\right)}{n-1} \end{array}$$ (2)

The component $\left(1-\frac{n}{N}\right)$ is the finite population correction, or *fpc*, and accounts for the reduction in variance caused by sampling without replacement and shrinking the remaining pool of articles to be sampled from.

To show an example of a specific calculation, in physics, the sample proportion of articles with data is $\frac{44}{50}=0.88.$ For physics, the fpc is computed by $(1-\frac{50}{467})=0.893$, and the variance of the estimate of the proportion of articles with data is computed as ${\hat{V}}_{data}=0.893*\frac{0.88*0.12}{49}=0.00192$. The standard error of the estimate is given by the square root of the variance, ${\hat{SE}}_{data}=\sqrt{0.00192}=0.0439$. A 95% confidence interval is given by ${\hat{p}}_{data}\pm 2.01*{\hat{SE}}_{data}$, where 2.01 is the.975 quantile for a t-distribution with 49 degress of freedom (*t*_.975,49_ = 2.01). We use the t-distribution in preference to the normal approximation because the sample size is relatively small. In the case of physics, the 95% confidence interval of the estimate of the proportion of all articles (in the top 10 journals) with data is 0.88 ± 0.088, or (0.792, 0.968).

### Proportion Estimates

Table 9 shows the proportion estimates by discipline and overall for the following parameters: the proportion of articles with data, the proportion of articles that reuse other data sources, the proportion of articles with original data, the proportion of articles that make their data available, and the proportion of articles with original data that make the data available. The method of computing the overall stratified sample estimates is discussed later. The numbers reported in parentheses are the proportions recomputed by dividing by the number of articles with data, not the total sample size. We are not only interested in the absolute proportions of the data categories, but in the characteristics of articles with data. It is more important for original research data to be made available than reused data, since the reused data is presumably already available from an alternative source. So, the proportion of original data articles that make their data available is perhaps the most important indicator of data sharing by discipline.

**Table 9:** Sample proportions/estimates of population proportions (n = 50 for each discipline, N = 200 overall).

*Discipline*	*Articles w/Data*	*Reused Data* ^†^	*Original Data* ^†^	*Available Data* ^†^	*Orig. Avail. Data*
Biology	0.580	0.020 (0.034)	0.560 (0.966)	0.240 (0.414)	0.429
Chemistry	0.860	0.160 (0.186)	0.700 (0.814)	0.060 (0.070)	0.057
Mathematics	0.380	0.100 (0.263)	0.280 (0.737)	0.120 (0.316)	0.286
Physics	0.880	0.360 (0.409)	0.520 (0.591)	0.080 (0.091)	0
Overall	0.760	0.147 (0.194)	0.613 (0.806)	0.104 (0.137)	0.130

^†^expressed as proportion of all articles (proportion of data articles in parentheses)

Table 10 reports additional parameters of interest: the proportion of review articles, the proportion of review articles with data, a ratio of the reused data articles to review articles with data, and the proportion of articles providing software code. The ratio of reused data articles to review articles with data reflects the fact that in three of the disciplines, the review articles were the major source of reused data. Only in mathematics were there several articles that reused data in the service of an original research project, as reflected by the ratio being larger than one. Also, mathematics is the one discipline with few review articles among its high-impact publications. This ratio is not computed for the overall population of articles, since it is not meaningful outside of the disciplinary context. The number and function of review articles differ dramatically by discipline.

**Table 10:** Sample proportions for additional variables.

*Discipline*	*Review Articles*	*Review Articles with Data*	*$\frac{Reused\;data\;articles}{Review\;articles\;with\;data}$*	*Software code*
Biology	0.260	0.077	1.000	0.100
Chemistry	0.300	0.533	1.000	0.020
Mathematics	0.020	1.000	5.000	0.080
Physics	0.400	0.850	0.944	0
Overall	0.276	0.519	-	0.040

It will be noted that no estimate of the proportion of articles with DOIs or other data citation is provided in the tables. This is because *none* of the articles examined cited data separately or provided unique identifiers. In a few journals, supplementary data files could be accessed by using the article’s DOI in combination with a postpended location marker, but this is not considered as full data citation according to the ideals of those promoting enhanced data citation. The percentage of articles made available by means other than the journal website is also not reported in the tables. The number of cases in which this occurred was small, and will be noted in the individual disciplinary discussions in the section on Disciplinary Differences.

### Estimates of Variance and Standard Error

The variance estimates, standard error estimates, and 95% confidence intervals on the population parameters are provided in Table 11. These are reported for articles with data (${\hat{V}}_{data}$, ${\hat{SE}}_{data}$), articles with reused data (${\hat{V}}_{reused}$, ${\hat{SE}}_{reused}$), articles with original data (${\hat{V}}_{original}$, ${\hat{SE}}_{original}$), articles with available data (${\hat{V}}_{available}$, ${\hat{SE}}_{available}$), and for the proportion of original data articles that make the data available (${\hat{V}}_{oad}$, ${\hat{SE}}_{oad}$). We do not compute these estimates for the secondary proportions in Table 10 partially because these sample sizes and proportions are too small for the confidence intervals to be of interest.

**Table 11:** Estimates of variance, standard error, and confidence intervals.

*variable*	*Biology*	*Chemistry*	*Mathematics*	*Physics*	*Overall*
fpc	0.943	0.981	0.879	0.893	-
${\hat{V}}_{data}$	0.00469	0.00241	0.00423	0.00192	0.00111
${\hat{SE}}_{data}$	0.0685	0.0491	0.0650	0.0439	0.0333
95% C.I. for data	(0.442, 0.718)	(0.761, 0.959)	(0.249, 0.511)	(0.792, 0.968)	(0.694, 0.826)
${\hat{V}}_{reused}$	0.00038	0.00269	0.00161	0.00420	0.00103
${\hat{SE}}_{reused}$	0.0194	0.0519	0.0402	0.0648	0.0322
95% C.I. for reused	(0, 0.059)	(0.056, 0.264)	(0.019, 0.181)	(0.230, 0.490)	(0.084, 0.210)
${\hat{V}}_{original}$	0.00474	0.00420	0.00362	0.00455	0.00177
${\hat{SE}}_{original}$	0.0689	0.0648	0.0601	0.0674	0.0421
95% C.I. for original	(0.422, 0.698)	(0.570, 0.830)	(0.159, 0.401)	(0.384, 0.656)	(0.530, 0.696)
${\hat{V}}_{available}$	0.00351	0.00112	0.00189	0.00134	0.00057
${\hat{SE}}_{available}$	0.0592	0.0336	0.0435	0.0367	0.0240
95% C.I. for available	(0.121, 0.359)	(0, 0.128)	(0.033, 0.207)	(0.006, 0.154)	(0.057, 0.151)
${\hat{V}}_{oad}$	0.00842	0.00224	0.01307	0	0.00140
${\hat{SE}}_{oad}$	0.0917	0.0473	0.1143	0	0.0374
95% C.I. for orig. avail. data	(0.241, 0.617)	(0, 0.153)	(0.039, 0.533)	(0)	(0.056, 0.204)

Most of the variances and confidence intervals are computed using Eq (2), as described above.

In order to compute the variance and confidence interval for the proportion of original data articles that make the data available, we must use a slightly different procedure, since the population sizes of articles with original data are themselves random variables. We use the technique of ratio estimation within population domains, where the domains are articles with data and articles without data. We compute $s_{oad}^{2}$ as the sample variance with the following formula, where *S*_*data*_ is the set of articles with original data, *y*_*i*_ is the indicator variable for available data, and ${\hat{p}}_{data}$ and *n*_*data*_ are as before.

$$\begin{array}{c} s_{oad}^{2}=\frac{\sum_{i\in S_{data}}{\left(y_{i}-{\hat{p}}_{data}\right)}^{2}}{n_{data}-1} \end{array}$$ (3)

Using this sample variance, we can compute ${\hat{V}}_{oad}$ with the following formula:

$$\begin{array}{c} {\hat{V}}_{oad}=(1-\frac{n}{N})\frac{n}{n_{data}^{2}}\frac{\left(n_{data}-1\right)s_{oad}^{2}}{n-1} \end{array}$$ (4)

**Fig 1:**
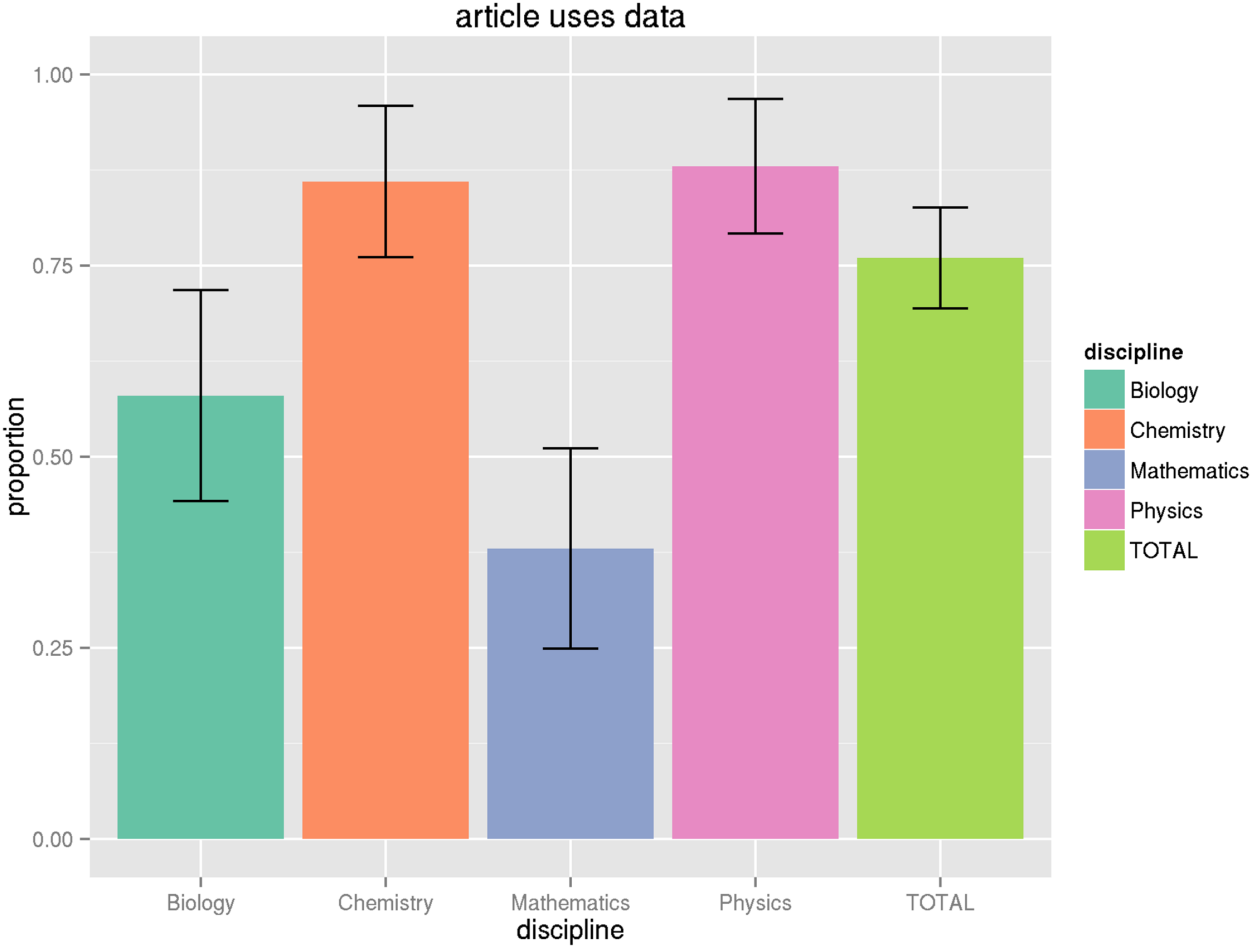
Proportion of articles using data. This graph shows the proportion of articles by discipline that make some use of data as part of the research, along with the confidence interval of this estimate for the general population, based on the sample size. See Tables 9 and 11 for numeric values.

**Fig 2:**
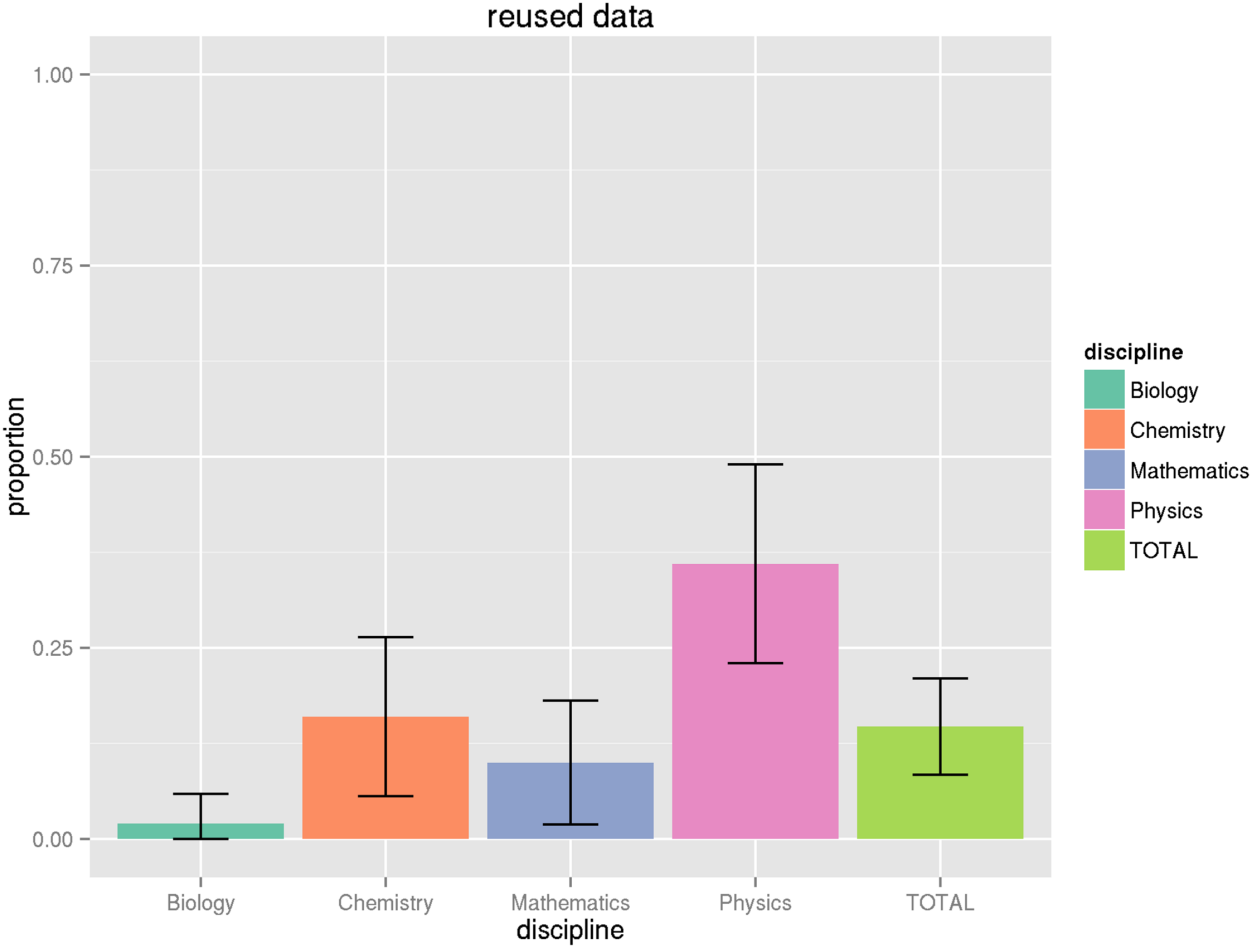
Proportion of articles with only reused data. This graph shows the proportion of articles with reused data, that is data taken from other studies and not original to the article in question, along with associated confidence intervals. See Tables 9 and 11 for numeric values.

**Fig 3:**
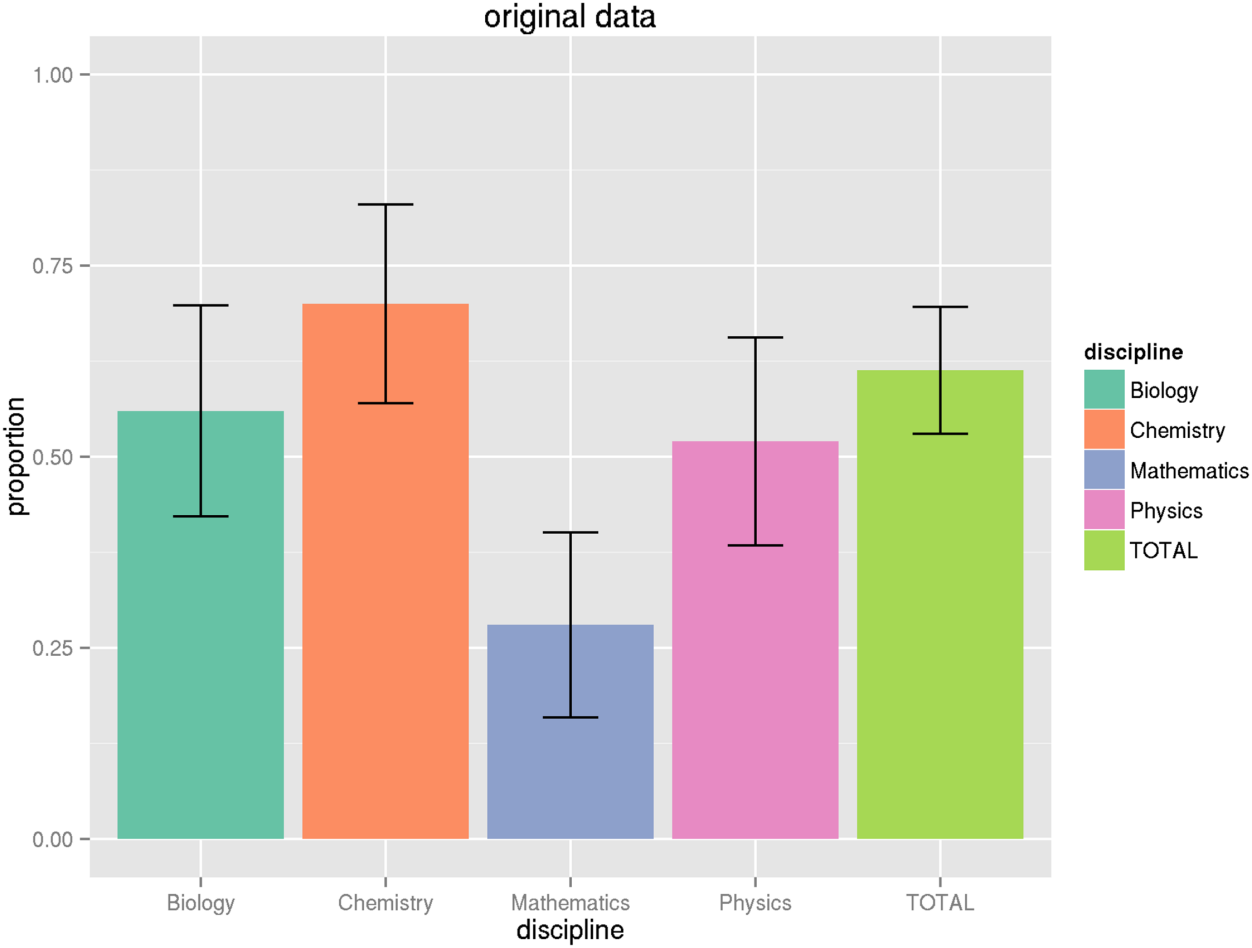
Proportion of articles with original data. This graph shows the proportion of articles by discipline with original data generated by the research described in the article, along with associated confidence intervals. See Tables 9 and 11 for numeric values.

**Fig 4:**
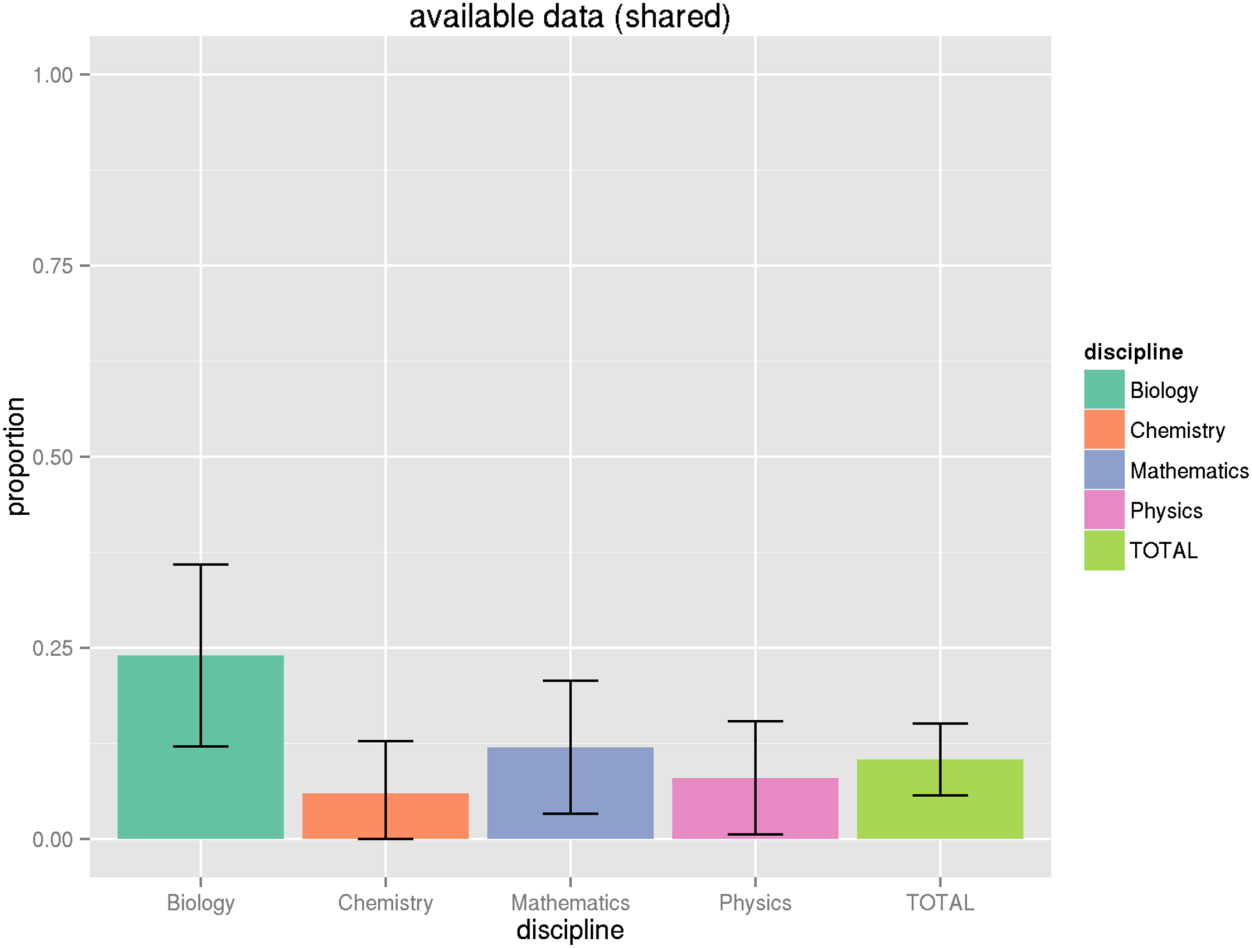
Proportion of articles that share data. This graph shows the proportion of all articles by discipline that share data, making it available to the reader via any indicated mechanism, along with associated confidence intervals. See Tables 9 and 11 for numeric values.

**Fig 5:**
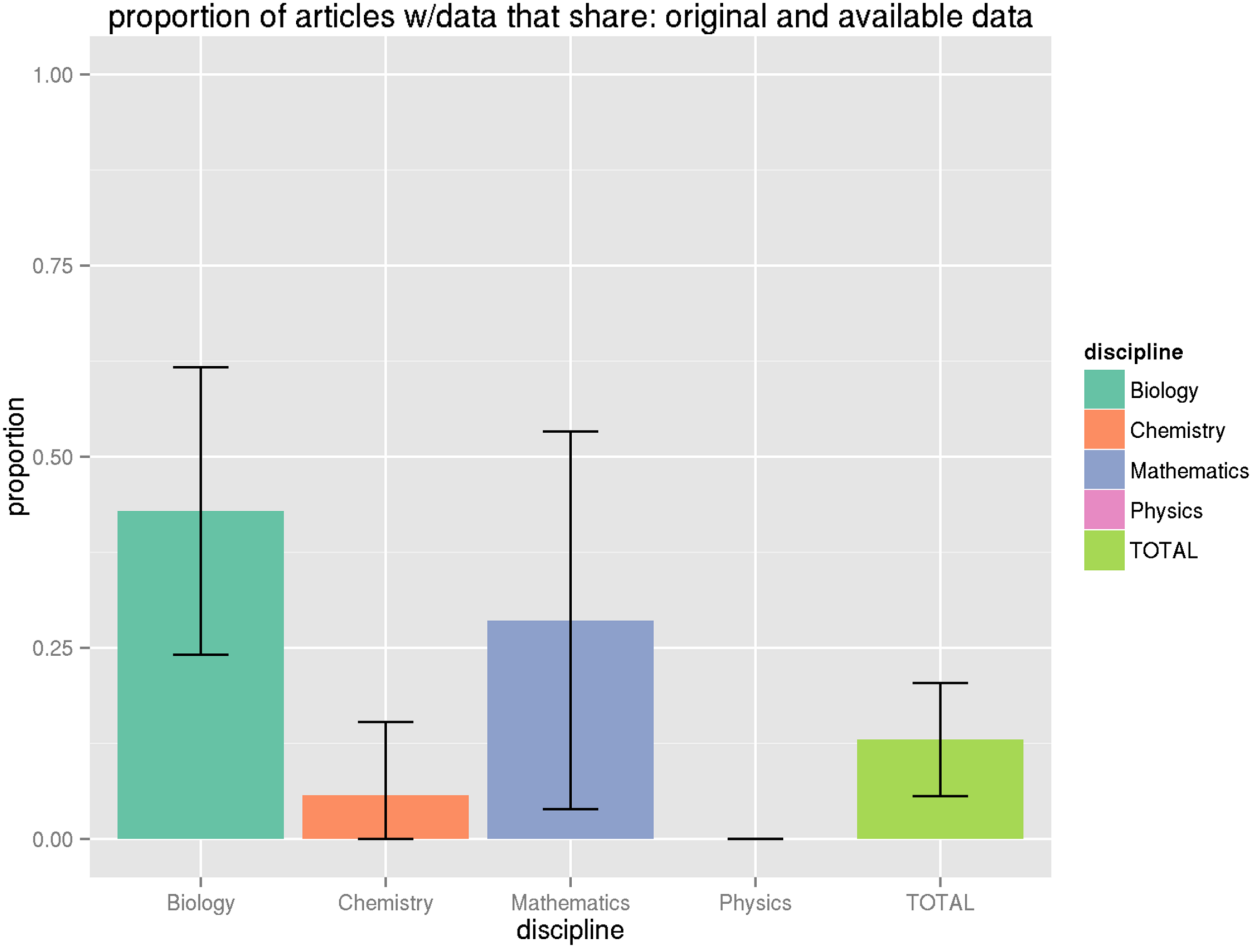
Proportion of original data articles that share data. This graph shows the proportion of articles by discipline that share data, among articles with original research data, along with associated confidence intervals. See Tables 9 and 11 for numeric values.

To illustrate with the numerical example of biology, $s_{oad}^{2}$ is $\frac{12*{\left(1-(3/7)\right)}^{2}+16*{\left(0-(3/7)\right)}^{2}}{27}=.254$, and ${\hat{V}}_{oad}=0.943\frac{50}{28^{2}}\frac{(27).254}{49}=0.084$. We use *t*_.975,27_ = 2.05, so the 95% confidence interval is slightly wider. The t-statistic used varies according to each discipline’s sample size of articles with data (Chemistry, *t*_.975,34_ = 2.03; Mathematics, *t*_.975,13_ = 2.16; Physics, *t*_.975,25_ = 2.06; and for the overall, *t*_.975,102_ = 1.98). However, we find that in most cases adjusting for the proportion within a domain does not greatly increase the variance, so we will not repeat this exercise for all of the proportions in parentheses reported in Table 9. See the Figures at the end of the article for graphical illustrations of the proportions and their associated 95% confidence intervals (Figs 1, 2, 3, 4 and 5).

### Disciplinary Differences

#### Biology

Biology top journals use data frequently, and data when used is almost always original to the article. However, only 42.9% of articles with original data make that data available. This is the highest rate among the four disciplines, but it is still far from a universal culture of data sharing. In fact, many of the articles examined provide only a few downloadable tables of data, so the estimate of 42.9% overstates the reality that far fewer articles make the complete research data package available.

Biology journals provide a variety of other data outputs. In the sample of 50 articles, five videos are available, and five articles make software code freely available (10% of the total). Biology is the only discipline to make widespread use of official external repositories for data sharing, with the Protein Data Bank (6 articles) (http://rscb.org), ProteomeXchange (2 articles) (http://www.proteomexchange.org), and Sequence Read Archive (2 articles) (http://www.ncbi.nlm.nih.gov/sra) represented. Five make use of figshare as integrated into the publisher’s website (http://figshare.com) to make tabular data downloadable. Additionally, 11 articles provide supplementary data only in PDF form, which were not counted as data since it is not in a form directly usable by another researcher. Only one article made data available through the author’s website.

#### Chemistry

In chemistry top journals, review articles use data about half of the time, but this is below the rate of data use for regular chemistry articles. Some review articles just summarize theory and stylized facts. Overall, 70% of articles use original data, the highest rate of the four disciplines. In fact, all of the sampled non-review articles use data, and all of that data is original. In terms of classification, if an article merely pictured a molecule, for example, it was not counted as a data article, but if a corpus of images was analyzed for patterns, it would be considered a data article. Some articles in chemistry presented images and graphics without supporting data alongside tables that summarized numerical chemical properties such as boiling point, molecular weight. These were considered articles with available data, although only part of the research data associated with the article was being shared.

Three articles of the 50 sampled articles made video available, and 16 articles put data into PDF formats, which did not count towards data availability. One of the articles contained software code that was reproduced only in PDF format, but was publicly available. Overall, chemistry articles made available only 5.7% of original data. Even considering the confidence interval, an upper bound on this estimate of data sharing is only 15.3%. So, in spite of the widespread use of data in top chemistry journals, the data is for the most part not made available. There are many graphs that visually summarize experimental results, but the underlying data tends not to be shared.

#### Mathematics

Many of the high-impact journals in mathematics report primarily theoretical results. The top 10 journals also include journals of mathematical statistics that may use data to illustrate applications of the methods developed in the article. While only 28% of all articles contained original data, 73.7% of the data articles used original data. The availability of data to readers was second highest of the four disciplines at 31.6%. In the sample of 50 articles, of the six articles with available data, four made the data freely available, while two kept the data behind the journal’s paywall. Two of the articles with freely available data used external websites to share the data. All of the 8% of articles that provided software code made the code freely available. Only one of the articles was a review article, a very different pattern than the other disciplines.

Several papers use simulated or synthetic data to illustrate functions and concepts. If these formed a substantial part of the argument of the paper, they were coded as data papers. The reasoning was that it would be desirable for a reader to access the data that these arguments were based on, so they could verify the results or test other theories with it. Since the number of math articles with data is relatively small, the estimate for the proportion of data articles that make the data available has a wide variance. The upper bound of the confidence interval on the percentage of papers that make data available is still only 53.3%.

#### Physics

The articles in the top ten physics journals are more likely to use data compared to the other three disciplines, with 88% of the articles using some data. Physics articles reuse other previously published data at a higher rate than the other disciplines, but the majority of articles still contain original data. Despite the prevalance of data, only 8% of the physics articles make data available, and of these four articles, only one has freely available data. The other three have data behind the paywall, only available to journal subscribers. By comparison, Mathematics has two articles with data behind the paywall, and Chemistry and Biology have one each. One of these paywalled articles has only a small portion of the data the article is based on available for download. None of these articles with available data were original research articles, so there is no data sharing in the area of greatest interest.

Two of the 50 articles sampled contain videos, but these are not the primary data sources for the articles. Three articles reproduced tables in PDF format only. As in chemistry journals, physics articles print many graphics that summarize the research data, but do not provide direct access to the underlying data. None of the sampled articles used links to outside websites or repositories, nor was any software code made available. In general, physics does little to share research data in a systematic way, at least in the top journals by impact factor.

### Combined stratified estimates

In order to obtain overall statistics on the data sharing practices in these four core science disciplines, we will combine the disciplinary estimates into one overall estimate using the techniques of stratified random sampling.

To estimate the overall proportions for articles for each variable under consideration, we will use the disciplinary estimates as our strata proportions, ${\hat{p}}_{h}$. We weight these estimates by the article counts for each of the four strata, and use formula (5) to estimate the overall proportion, ${\hat{p}}_{str}$. In the equations below, *n*_*h*_ refer to the strata sample size, and *N*_*h*_ refers to the strata population, here the total number of articles published in the discipline. Weighting the strata proportions by the number of articles in the discipline is in this case equivalent to other common technique of applying weights at the article level prior to averaging. Here we use the following formula:

$$\begin{array}{c} {\hat{p}}_{str}=\sum_{h=1}^{H}\frac{N_{h}}{N}{\hat{p}}_{h} \end{array}$$ (5)

As a numerical example, to compute the proportion of articles with data, we use *N* = 883 + 2606 + 414 + 467 = 4370, and so ${\hat{p}}_{str}=\frac{883}{4370}0.58+\frac{2606}{4370}0.86+\frac{414}{4370}0.38+\frac{467}{4370}0.88=0.76$. Table 9 reports the combined estimates of proportions in the *Overall* column.

When combining strata, the variance is estimated by using Eq (6).

$$\begin{array}{c} {\hat{V}}_{str}\left({\hat{p}}_{str}\right)=\sum_{h=1}^{H}(1-\frac{n_{h}}{N_{h}}){\left(\frac{N_{h}}{N}\right)}^{2}\frac{{\hat{p}}_{h}\left(1-{\hat{p}}_{h}\right)}{n_{h}-1} \end{array}$$ (6)

Note that within each strata, this equation is the same as the variance reported for individual disciplines in Table 11, multiplied by ${\left(\frac{N_{h}}{N}\right)}^{2}$. The confidence interval multiplies the standard error by the t-distribution critical value with *n* − *H* degrees of freedom, or 200-4 = 196 degrees of freedom in this case (*t*_.975,196_ = 1.97). Table 11 reports the combined variance and standard error estimates in the *Overall* column, along with the 95% confidence intervals they generate on the proportion estimates. As with the individual disciplines, the estimate of the variance for overall original available data is computed using ratio estimation within a domain using Eq (4). See the Supplementary Information for the spreadsheet containing this calculation. In this case, the confidence interval is based on a t-statistic of 1.98 on 102 degrees of freedom (*t*_.975,102_ = 1.98).

The values of the combined proportions most closely track the chemistry proportions, since chemistry articles dominate the sample. The benefits of combining the data lie primarily in the narrower confidence intervals generated on the proportions. Overall, 76% of articles use data, 61.3% generate original data (80.6% of the data articles), but only 10.4% of articles make data available. Only 13% of articles with original data make at least some of that data available in machine-actionable form to the reader. This 13% rate is not very different from the 9% found by Alsheikh-Ali et. al. [27], suggesting only slow progress in the growth of data sharing.

## Discussion

It is important to keep in mind what this study demonstrates and where its limits lie. It is a statistically valid portrait of a set of the top 40 journals in four disciplines as ranked by impact factor. The combined estimates describe the total population at the article level. To illustrate, if we put all articles (and reviews) published by these 40 journals in 2014 into a pile and randomly selected one, our expectation that it would use data is 76%, that it would have data available would be 10.4%, and so on. The confidence intervals as constructed are valid when applied to this population, but cannot be easily generalized to other contexts. This study is suggestive about data sharing practices in these disciplines as a whole, if we believe that the high-impact factor journals are influential and lead the disciplines’ scholarly practices. Ultimately, however, this study provides no information on the data sharing behavior in the vast majority of journals in these disciplines, and of course no information at all on other disciplines. It does, however, allow direct comparisons among the four disciplines, something not done in prior work.

The presence and behavior of review articles, primarily appearing in the leading review journals, is another issue. These articles do not use original, newly generated research data, but most often describe and draw on the data from many prior studies to establish their summary views. While it would also be desirable if this data was well-cited and shared, it is of greater importance for ongoing research that the truly novel data in original articles be shared at the time of its creation. Future studies may wish to exclude review journals or consider them as an entirely separate category.

Data availability policies are another factor which may influence data sharing behaviors. All *Nature* journals require authors “to make materials, data, code and associated protocols promptly avaialable to readers without undue qualifications” (http://www.nature.com/authors/policies/availability.html). Among the 40 journals in this study, this includes *Nature Biotechnology, Nature Chemical Biology, Nature Chemistry, Nature Methods, Nature Photonics*, and *Nature Physics*. *Nature Protocols* does not really ask for data due to the nature of the protocols described. *Nature* also encourages parallel publication of significant data sets in the journal *Scientific Data*.

Other journals that ask that data be made available are *Physiological Reviews, Journal of the Royal Statistical Society Series B (Statistical Methodology)*, and *ACS Nano*. *PLOS Biology* implemented its data availability policy (http://journals.plos.org/plosbiology/s/data-availability) on March 3, 2014.

Despite the presence or phasing in of these policies, the availability of data has not grown much in the top journals. However, this is an area deserving of more detailed study to determine how many articles are compliant with policy and how influential these policies have been. The present study was not designed with those goals in mind, so it is only suggestive on this topic.

Some surprising results emerge from the sample. Data citation, although widely discussed as an important and growing practice in scientific research, has not reached the high-impact journals in any of these disciplines as of 2014. Separate DOIs for data resources are not used, and direct links to data are rare. More typical, and perhaps disturbing, is the often encountered loose style of reference where an author, without using an endnote or reference, may state in the text of the article “I used the Panel Study of Income Dynamics” [or some other data resource] without providing any precision on the location of the data, date of access, components of the data used, or any other detail. Sharing of software code is also rare, inhibiting reproducibility of results.

Data sharing itself is not prevalent at all in the top physics and chemistry journals. There is more data sharing in the top journals in mathematics and biology, but even here it is not as widespread as could be hoped. The most conservative statement that can be made, by taking the maximum upper bound of the 95% confidence interval on the proportion estimates, is that not more than 61.7% of biology articles in the top 10 journals that use data take any steps to make the data available to readers. This is in the case of biology, but the estimates in other disciplines are far lower. This “upper bound” on data sharing is only 20.4% across all top 40 journals. Also, these upper bounds overstate the extent of data sharing in an important way, since many articles qualified as sharing data when only a small portion of tabular data was available for download. Very few articles in any discipline included links to the kind of large-scale original raw data envisioned by data sharing advocates. This is similar to the findings of Nicholson and Bennett, referenced earlier, that none of the dissertations in their sample provided complete original datasets [26]. The large bundle of raw data is rarely found in the top 40 high-impact journals, at least in 2014 in biology, chemistry, mathematics, and physics. Federal mandates for data sharing may increase these rates in the future, but as of 2014 this impact was not being felt yet.

One hypothesis is that greater data sharing and citation may be occurring at less highly-ranked journals that are more fluid in their practices. Journals that focus on publishing a high volume of relevant results rather than selectivity may also behave differently, although even journals created with an emphasis on openness such as *PLoS Biology* do not yet exhibit advanced data sharing behaviors. Regardless of these potential explanations, the current study provides clear evidence on the practices in the top 40 journals sampled.

The confidence intervals on the proportion estimates are not narrow, given the small sample sizes within each discipline. In spite of this, most of the confidence intervals are widely separated and often do not overlap at all. So we can use the estimates in Table 11 to make unambiguous and reliable statements such as “chemistry articles use data more often than mathematics articles” and “biology data articles share their data more often than physics data articles”, with the caveat that we are always discussing articles appearing in the top 10 high-impact journals in each discipline. We should also remember that the results are heavily influenced by the journals with a high volume of articles, such as *ACS Nano*. The other caveat we should keep in mind is that this study is based on 2014 articles only, and that earlier or later time periods may have different patterns. With these caveats noted, the differing proportions and confidence intervals are clear evidence of disciplinary differences in data sharing behavior.

It is important to keep in mind the limitations of this study and the nature of its design. Due to time and resource constraints, only a limited number of articles could be sampled and studied closely for the presence of data. This sample size is sufficient to draw some conclusions about the disciplinary differences among the top journals as discussed above, and there is no *a priori* reason to think that the sample is biased or unrepresentative of the disciplines. However, those with more discipline-specific knowledge may wish to see more detail about specific journals or to ensure their balanced representation if particular journals are known to have different patterns. For example, given the dominant role of the high volume of *ACS Nano* articles, it would be good to know chemists’ opinions of whether this journal is typical or atypical of data-sharing patterns, and whether it makes sense to weight it in proportion to the number of articles published.

A larger sample size would improve the precision of the estimates, and a design that was also stratified at the individual journal level would allow for more specific comparisons at the cost of some complexity in sample design and computation. It would also be interesting to make comparisons over time by sampling several years of articles.

In that sense, the current study could be viewed as an exploratory study establishing some initial findings that could be refined by further work. By relying on objective criteria for sample construction and methodology, however, this study does provide a factual baseline for other potential studies using expert judgment to refine the sampling and population to be studied.

This research could be extended in several different ways. The confidence intervals are proportional to the square root of the sample size, so if one wanted to double the precision of the estimates, a sample of four times the size could be constructed. The same sampling techniques could be applied to other disciplines such as engineering and medicine, or to a wider range of journals, or used to provide more detailed estimates at the level of the individual journal, by using the journals as strata as indicated above. Those more familiar with disciplinary practices may delve more deeply into the individual disciplines of biology, chemistry, mathematics, and physics to reveal and explain more of the reasons behind the data citation practices observed.
